# How many marker loci are necessary? Analysis of dominant marker data sets using two popular population genetic algorithms

**DOI:** 10.1002/ece3.725

**Published:** 2013-08-28

**Authors:** Michael F Nelson, Neil O Anderson

**Affiliations:** Department of Horticultural Science, University of Minnesota1970 Folwell Ave, Saint Paul, Minnesota, 55108

**Keywords:** AFLPs, AMOVA, Invasive species, ISSRs, *Phalaris arundinacea*, sample size, STRUCTURE

## Abstract

The number of marker loci required to answer a given research question satisfactorily is especially important for dominant markers since they have a lower information content than co-dominant marker systems. In this study, we used simulated dominant marker data sets to determine the number of dominant marker loci needed to obtain satisfactory results from two popular population genetic analyses: STRUCTURE and AMOVA (analysis of molecular variance). Factors such as migration, level of population differentiation, and unequal sampling were varied in the data sets to mirror a range of realistic research scenarios. AMOVA performed well under all scenarios with a modest quantity of markers while STRUCTURE required a greater number, especially when populations were closely related. The popular ΔK method of determining the number of genetically distinct groups worked well when sampling was balanced, but underestimated the true number of groups with unbalanced sampling. These results provide a window through which to interpret previous work with dominant markers and we provide a protocol for determining the number of markers needed for future dominant marker studies.

## Introduction

Dominant markers systems such as Amplified Fragment Length Polymorphisms (AFLPs) (Vos et al. 1995) and Inter Simple Sequence Repeats (ISSRs) (Zietkiewicz et al. 1994) are commonly used to characterize population genetic structure. There is little initial time and effort required to develop primer sets as with Simple Sequence Repeats (SSRs) (Nybom 2004) and their relatively inexpensive cost makes them ideally suited to studies of non model organisms. As next-generation sequencing technology matures and becomes less expensive, techniques such as restriction-site-associated DNA (RAD) tags (Baird et al. 2008) will likely supplant the use of dominant marker systems. However, there exists a sizeable body of literature on these methods and they are still widely used.

Sufficient quantities of marker loci and individuals sampled are key to measure population parameters accurately (Bonin et al. 2007). An important question when planning an experiment using dominant markers is: “What is the minimum number of marker loci sufficient to address the research objective?” An additional factor is the number of individuals sampled per population. The answers depend on many factors including the level of neutral genetic diversity, gene flow, the level of population differentiation, and the particular research question (Wolfe et al. 1998; Schmidt and Jensen 2000; Hollingsworth and Ennos 2004; Singh et al. 2006). When beginning a dominant marker study, an initial screen of multiple primers may provide a number of polymorphic polymerase chain reaction (PCR) fragments. The final number of markers used may, therefore, be based primarily on convenience or chance rather than on a data-generated minimum number of marker loci required to address the research goals. Although such an initial screen may yield a number of scoreable marker loci, this number may not be sufficient to address a particular research objective such as finding the number of genetically distinct groups within a metapopulation. Conversely, sampling more markers than necessary for a given set of populations can be inefficient and result in unnecessary expense (Cavers et al. 2005).

Some guidelines exist for the number of individuals to sample per population and the recommended number of markers to use in the context of specific organisms such as spatial genetic structure in tree populations (Cavers et al. 2005) and sampling diversity in wild relatives of wheat (Singh et al. 2006). A starting point of 200 markers, with additional loci added as needed to address the specific research question, has been recommended (Bonin et al. 2007). In typical AFLP studies, anywhere from several hundred to over 1000 polymorphic marker loci have been used (Schmidt and Jensen 2000; Bezault et al. 2011). Many ISSR studies have used between 50 and several hundred loci (Wolfe et al. 1998; Meekins et al. 2001). Nybom (2004) found that ISSR studies used an average of 55 marker loci, while AFLP studies averaged 238.

A recent molecular study of the widespread invasive grass, *Phalaris arundinacea* L. (Nelson et al. 2013), which is native to Europe and North America (Merigliano and Lesica 1998; Galatowitsch et al. 1999; Jakubowski et al. 2013) with repeated introductions of European genotypes to N. America, used 90 ISSR markers to characterize the population structure of North American and European populations. This study used this species as a model organism from which to simulate data sets to test the performance of two commonly used population genetics analyses to determine the minimum number of loci required. In the ISSR study (Nelson et al. 2013), analysis of molecular variance (AMOVA) (Excoffier et al. 1992) was used to examine the degree of population genetic differentiation and STRUCTURE (Pritchard et al. 2000) was used to detect genetically distinct groups.

During work on the molecular study of *P. arundinacea* using ISSRs (Nelson et al. 2013), the question of how many marker loci were needed to address the research questions arose frequently. Using simulated data can be a useful method to assess the power of analyses with a given number of samples and loci (Balloux 2001). With simulated data sets, factors such as the level of neutral variation, population differentiation, migration, and unequal sample sizes can be experimentally varied to test the performance of selected analyses under a range of biologically relevant scenarios. The main objective of this study was to determine the minimum number of dominant marker loci required to obtain results that reflect the true population structure from two commonly used population genetics analyses, Analysis of Molecular Variance (AMOVA; Excoffier et al. 1992) and STRUCTURE (Pritchard et al. 2000; Falush et al. 2007), using simulated data sets. Secondary objectives were to observe if the minimum number of loci required varies with small sample sizes, to assess the ability of STRUCTURE to detect admixed individuals over time, and to provide a reference through which to interpret previous and current dominant marker studies in terms of adequacy of sampling and number of polymorphic loci.

## Material and Methods

### Model population structure and sampling

**Figure 1:**
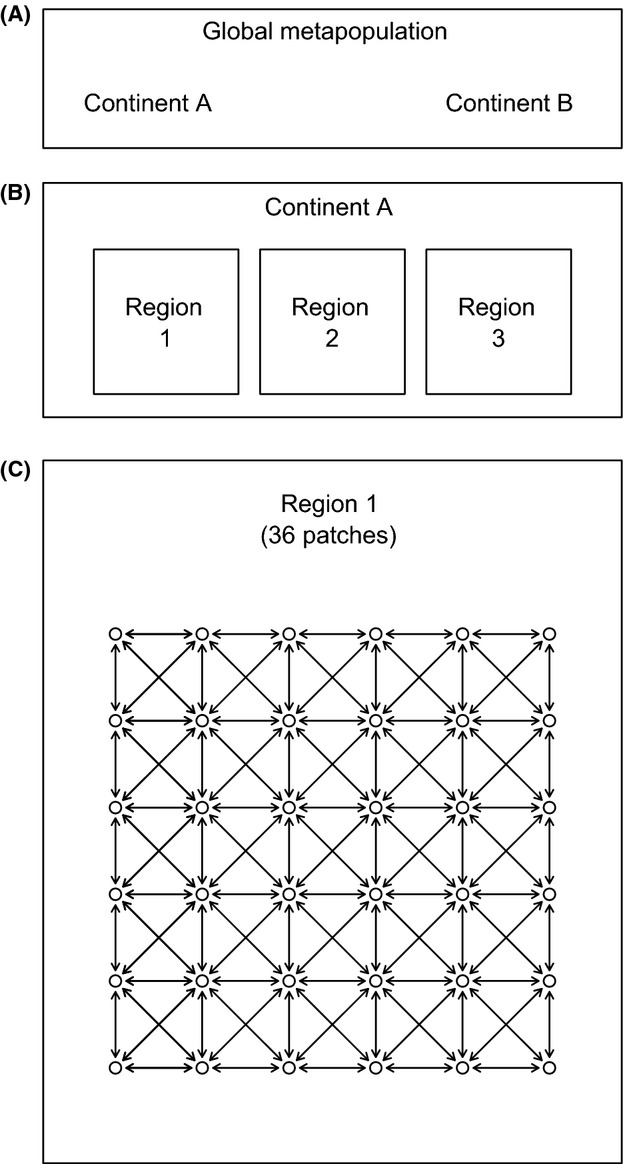
The global metapopulation of a simulated set of plant populations, 553 divided (A) into two continents A and B, (B) each with three regions (1–3). Each region consisted of a square lattice of 36 patches (C). Regions 1–3 are within continent A, regions 4–6 (not shown) are within continent B. The arrangement of patches was the same in each region. Patches within regions were randomly selected for sampling. Circles in (C) indicate patches, while arrows indicate possible migration routes. Migration was possible between neighboring patches (eight for interior patches, three for corner patches, and five for edge patches; see text).

To simulate real populations of a widespread organism such as *P. arundinacea*, global metapopulations were simulated comprising two continents (representing for example N. America and Europe), each of which had three regions. Regions were further divided into 36 patches (Fig. 1). The regions represented geographically isolated areas within a continent, for example the Pacific Northwest, the American Midwest, and New England in N. America; or France, Sweden, and the Czech Republic in Europe. A square number of patches was used to have a convenient square lattice for migration. The carrying capacity of each patch was set to 1000 individuals.

Three models were created to address questions of unequal sampling and migration. Model A, with equal sampling, was the simplest model with six patches randomly selected from each of the six regions for a total of 36 sampled patches. Model B introduced unequal sampling among regions and between continents with regions one and two sampling one patch each, regions three and four sampling five patches each, and regions five and six each sampling 12 patches. Model C utilized the equal sampling scheme of Model A, but introduced among-region migration. To test the effect of sample size on the analyses, two series of data sets were created, one with 10 individuals sampled from each selected patch and one with five individuals sampled.

### Simulated genomes

**Figure 2:**
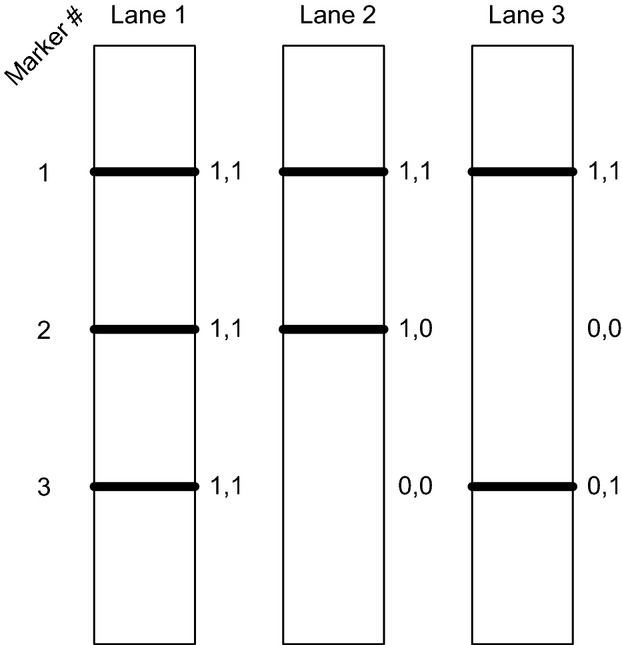
Simulated dominant markers scored as if they represented bands on a gel (present [+] or absent [−]). The genotype of each sample at each marker locus is located to the right of the lane. Dominant homozygotes and heterozygotes (genotypes 11 and 01/10) appear as bands on the gel, for example, marker #1 in lane one (a dominant homozygote). Recessive homozygotes are represented by a blank space, for example marker #3 in lane two. Heterozygotes were scored identically to dominant homozygotes, for example marker #2 in lane 2.

The dominant cytotype of *P. arundinacea* is allotetraploid with 28 chromosomes (McWilliam and Neal-Smith 1962), potentially with diploid-like inheritance. To simplify the creation of data sets, all individuals were simulated with diploid genomes consisting of 14 chromosomes (2*n* = 2*x* = 14). Each of the chromosomes was assigned a length of 120 centimorgans (cM). The value of 120 cM allowed for pairs of marker loci on a single chromosome to be linked (less than 50 cM apart) or unlinked (greater than 50 cM apart). To simulate dominant markers such as AFLPs or ISSRs, 1000 biallelic loci were randomly assigned to positions on the simulated chromosomes. One thousand total marker loci were used because many AFLP and ISSR studies use fewer than 1000 markers (Nybom 2004). The two alleles for each marker locus were designated “0” and “1” with “1” being the dominant allele. As heterozygotes and homozygous dominants are not distinguished in dominant marker systems, the heterozygous (0,1 or 1,0) and homozygous dominant (1,1) genotypes were scored as present (+), while the homozygous recessive (0,0) was scored as absent (−), similar to bands on a gel (Fig. 2).

The *P. arundinacea* study of Nelson et al. (2013) utilized 90 ISSR markers. Many ISSR studies have used fewer (Culley and Wolfe 2001; Meekins et al. 2001). Thus, to capture the range of marker numbers typically used, data sets comprising 30, 45, 90, 200, 500, and 1000 marker loci were subsampled from the simulated genome data.

### Allele frequencies

**Figure 3:**
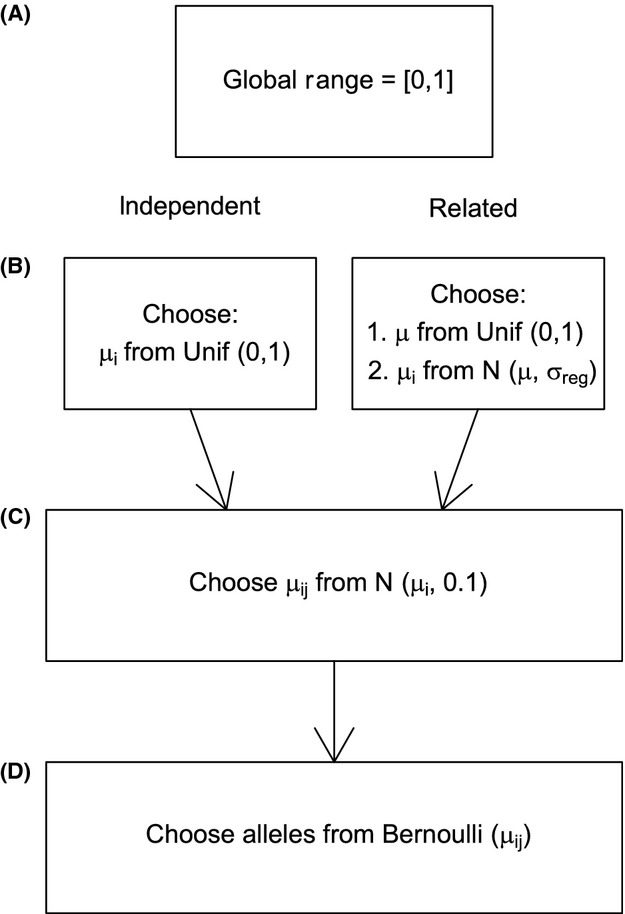
Flowchart of the simulated allele assignment process with the (A) global allele frequency range [0, 1] for patch *j* within region *i;* (B) regional allele frequencies randomly drawn where the choice of regional allele frequencies depends on whether region-level allele frequencies are independent or related. if independent, then the six regional allele frequencies are chosen from Unif(0, 1), If related, they are chosen from a Normal distribution; (C) patch-level allele frequencies, drawn normally with *μ* = *μ*_*i*_; (D) two alleles for each genotype in patch *j* are independent Bernoulli random variables.

To set the initial allele frequencies within each model, a hierarchical method, inspired by the region and population hierarchies used in AMOVA (Excoffier et al. 1992), was used. At the region level, allele frequencies were either independent or related. A standard deviation parameter *σ*_reg_, was used to account for the similarity among related regions (Fig. 3). To simulate different levels of relatedness among regions, *σ*_reg_ had four levels: 0.05, 0.10, 0.15, 0.20. The low values flank the actual ranges of 0.05–0.08 calculated from Nelson et al. (2013), while the higher values, and the independent case, represent scenarios with more strongly differentiated regions.

For data sets with related regions, a global dominant allele frequency (*μ*) for each marker locus was drawn from a uniform distribution on the interval [0, 1]. Next, six region-level allele frequencies (*μ*_*i*_, *i* = 1, …, 6, *i* = region number) were drawn normally from 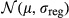. For data sets with independent regions, the six values of *μ*_*i*_ were drawn from a uniform distribution, Unif(0, 1). To assign allele frequencies to patches within region *i*, 36 allele frequencies (*μ*_*ij*_, *j* = 1, …, 36) were drawn from 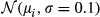. For all normally distributed parameters, allele frequency values outside the range [0,1] were truncated to 0 (lost) or 1 (fixed). The 0 and 1 alleles were assigned to each of the 1000 genotypes within a patch using Bernoulli trials with *P* = *μ*_*ij*_.

### Migration

Two types of migration were used within the models to simulate dispersal of propagules (seeds, spores, or vegetative propagules) or individuals in the case of animals. Background, or within-region, migration occurred in Models A–C, while among-region migration was restricted to Model C, in which intercontinental migration occurred. To simulate background migration, individuals were allowed to migrate between their patch and the immediate neighbors using a two-dimensional stepping stone model (Kimura and Weiss 1964; Fig. 1C). Each region was arranged as a square lattice of 36 patches so that genotypes could migrate to any one of their eight neighbors (five neighbors for edge patches and three neighbors for corner patches). Data sets for each model were created with the proportion of patch migrants set to 0 (no background migration) and 0.1 (background migration).

**Figure 4:**
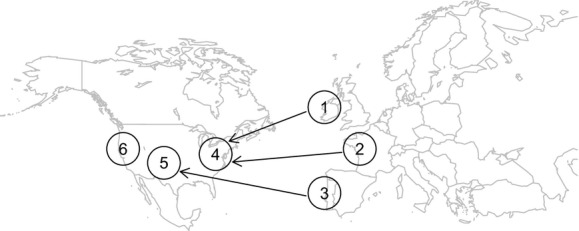
Model C simulated regions (circles) and migration paths (arrows), with among-region migration schemes based on the movement of *Phalaris arundinacea* genotypes from Europe to N. America.

The among-region migration scheme (Model C) was constructed to mirror the human-mediated dispersal of *P. arundinacea* (Fig. 4), which is native to N. America and Europe (Merigliano and Lesica 1998; Jakubowski et al. 2013), with repeated introductions of European genotypes to N. America (Galatowitsch et al. 1999). To model a scenario of multiple introductions, 18 patches in region four were randomly selected to receive immigrants from 18 randomly chosen region one patches. Similarly, 18 patches were randomly selected to receive immigrants from region two. A given patch in region four could receive no immigrants, immigrants from region one, immigrants from region two, or immigrants from both. A single introduction event was modeled by having immigrants from 18 patches in region three randomly migrate to 18 patches in region five. Model C among-region migrations occurred between generations one and two.

### Simulated populations

In all models, metapopulations were created which consisted of the six regions, each with 36 patches having carrying capacities of 1000 individuals. For Models A and B, a common set of 10 metapopulations was created, one metapopulation for each combination of *σ*_reg_ and background migration level. For Model A, six patches were randomly sampled from each of the six regions. Each metapopulation simulated was run for 150 generations. For Model B, the sampled patches were unequally distributed between regions as described above. Two Model C metapopulations (with and without background migration) were created with independent regions. To observe the effects of among-region migration over time, Model C individuals were sampled from generations 1, 2, 50, 100, and 150.

### Forward simulations

The above metapopulations were evolved using the forward-simulator quantiNemo (Neuenschwander et al. 2008). The breeding system was modeled with individuals acting as randomly mating hermaphrodites to approximate the breeding system of *P. arundinacea,* a highly self-incompatible wind-pollinated species (Weimarck 1968). To simulate among-region migration events (Model C), randomly selected individuals were sampled from the emigrant patches and added to the immigrant patches in generation two.

### Analyses

AMOVA (Excoffier et al. 1992) was used to partition the genetic variance at the among-region, among-patch (within-region), and within-patch levels. The models' regions and patches corresponded to the region and population levels in AMOVA. AMOVAs were calculated using package “ade4” (Dray and Dufour 2007) in R (R Development Core Team 2011). Population genetic differentiation was measured using Φ statistics (Excoffier et al. 1992) based on 999 permutations. AMOVAs were performed on the data sets with 30, 45, 90, 200, 500, and 1000 marker loci. To create a reference against which to compare the performance of AMOVA, reference analyses were performed on 1000-marker data sets with 150 individuals sampled from the selected patches.

To evaluate the performance of STRUCTURE (version2.3.2, Pritchard et al. 2000; Falush et al. 2007), a popular Bayesian clustering tool, and all sampled datasets were analyzed. The STRUCTURE algorithm assumes Hardy-Weinberg equilibrium within populations and minimizes the disequilibrium by arranging individuals into populations (Pritchard et al. 2000). Ideally after a suitable number of burnin (initial permutations before data are recorded) and Markov Chain Monte Carlo (MCMC, data-generating permutations) repetitions, the genotypes are proportionally assigned to K (specified by the user) groups. Each individual is assigned a coefficient associated with each of the K groups (all summing to 1). A coefficient close to 1 for a particular group indicates that the individual is highly likely to have originated from the group in question, while approximately equal values associated with multiple groups may indicate either admixture or the lack of a sufficient pattern in the data for the algorithm to resolve that individual's true group membership.

The performance of the STRUCTURE algorithm was evaluated by examining bar plots of the K coefficients for K = 6 (the true number of distinct groups). In the bar plots, each coefficient was assigned a different color. If individuals within regions were assigned the same color on the bar plot and all regions were distinctly separated, the algorithm was considered to have correctly identified groups. If individuals had nearly equal parts of each shade or if regions were not clearly differentiated, the algorithm did not correctly identify groups.

All STRUCTURE runs were performed with the following program settings: 100,000 burnin and MCMC repetitions, admixture model, and allele frequencies correlated. To evaluate the performance of STRUCTURE's grouping algorithm, bar plots of all sampled genotypes were analyzed for all models. To visualize the effects on the analysis of migration over time for Model C, one simulation at K = 6 was run on the 200 marker loci data sampled from generations one, two, 50, 100, and 150.

To determine the most likely number of clusters, we used the methods of Evanno et al. (2005) for models A and B. Evanno et al. (2005) created the ad hoc statistic, ΔK, which is, based on second-order derivatives of the log likelihood scores produced by STRUCTURE. To determine the most likely number of distinct groups in the data, a number of simulations are performed over a range of K values. A peak of ΔK at a particular value of K indicates the most likely true value for K, with the height of the peak indicating the level of confidence. To determine ΔK, STRUCTURE simulations were run with K from one to eight using five repetitions at each level of K on the generation 150 data for Models A and B.

## Results

### AMOVA

In the reference data sets, three major trends were apparent. First, as the region-level allele frequencies went from independent to highly related (region-level allele frequencies independent to *σ*_reg_ = 0.05) the proportion of among-region variance decreased. For example, in data sets without background migration the among-region variance decreased from 19.1% to 1.2% of the total (Table 1A). Second, as the region-level allele frequencies went from independent to highly related (independent to *σ*_reg_ = 0.05) the percentage of variance attributed to the within-patch level increased. For instance, in data sets with background migration the within-patch variance increased from 78.7% to 97.3% of the total (Table 1A). Finally, when background migration occurred, the among-patch variance proportions were reduced. For example, with *σ*_reg_ = 0.15 the among-patch variance was 13.2% of the total without background migration versus 0.6% of the total with background migration (Table 1A).

**Table 1:** AMOVA partitioning of genetic variance in a simulated study of the (A) genetic variance among regions, among populations, and within populations of the reference populations (1000 marker loci, 150 individuals sampled per patch); (B, C) equal sampling (Model A) with five and 10 individuals sampled per patch, respectively; (D, E) unequal sampling (Model B) with five and 10 individuals sampled per patch, respectively

*σ*_reg_	No. of markers	No background migration			Background migration		
	
		% Among region	% Among patch	% Within patch	% Among region	% Among patch	% Within patch
(A)							
independent	1000	19.1	11.9	69.0	20.8	0.5	78.7
0.20	1000	18.2	12.4	69.4	20.1	0.6	79.3
0.15	1000	11.6	13.2	75.2	12.6	0.6	86.7
0.10	1000	5.7	14.1	80.2	6.6	0.6	92.8
0.05	1000	1.2	14.5	84.3	2.1	0.6	97.3
(B)							
independent	30	20.9	13.1	66.0	17.6	-0.2	82.6
0.2	30	16.5	14.6	68.9	23.3	1.7	75.0
0.15	30	8.8	15.5	75.7	13.1	0.1	86.8
0.1	30	5.3	13.2	81.5	6.0	1.7	92.3
0.05	30	3.3	16.1	80.6	2.7	-0.7	98.0
independent	45	20.3	10.4	69.3	19.2	1.5	79.3
0.2	45	15.3	15.0	69.8	22.1	0.6	77.4
0.15	45	10.6	13.4	76.0	13.3	1.1	85.6
0.1	45	4.7	14.7	80.5	6.9	2.0	91.1
0.05	45	1.0	15.7	83.2	1.1	2.0	96.9
independent	90	20.4	12.9	66.7	19.3	0.7	79.9
0.2	90	16.6	13.8	69.6	21.5	-0.4	78.9
0.15	90	10.9	14.3	74.8	13.6	0.5	86.0
0.1	90	5.2	13.6	81.3	6.3	1.5	92.3
0.05	90	1.4	16.1	82.4	2.0	0.0	97.9
independent	200	19.0	12.7	68.3	19.7	0.3	80.0
0.2	200	19.1	12.8	68.0	20.8	0.8	78.4
0.15	200	11.1	14.6	74.3	12.6	1.0	86.4
0.1	200	4.8	13.9	81.2	7.0	1.0	92.1
0.05	200	1.9	15.5	82.6	1.4	1.5	97.1
independent	500	19.1	12.0	68.9	19.9	0.4	79.7
0.2	500	17.8	12.8	69.4	19.8	0.9	79.3
0.15	500	11.3	13.6	75.1	13.4	0.5	86.1
0.1	500	5.1	14.4	80.5	6.6	0.3	93.0
0.05	500	1.5	15.0	83.6	1.8	0.5	97.7
independent	1000	19.1	12.2	68.8	19.8	0.6	79.6
0.2	1000	17.9	13.1	69.0	20.3	0.8	78.9
0.15	1000	11.3	13.5	75.2	13.1	0.7	86.2
0.1	1000	5.1	14.5	80.4	6.1	0.3	93.6
0.05	1000	1.2	15.0	83.8	2.0	0.8	97.3
(C)							
independent	30	21.5	11.4	67.1	18.1	1.1	80.8
0.20	30	17.8	12.9	69.2	23.3	-0.2	76.8
0.15	30	10.2	13.6	76.3	12.8	0.7	86.5
0.10	30	4.2	13.6	82.3	7.3	1.4	91.3
0.05	30	1.0	15.6	83.4	1.5	0.9	97.6
independent	45	19.6	12.4	68.0	19.3	1.4	79.3
0.20	45	15.6	14.6	69.9	21.2	0.6	78.1
0.15	45	9.2	13.5	77.3	13.2	0.8	86.0
0.10	45	5.5	14.1	80.5	6.6	-0.2	93.5
0.05	45	1.6	15.8	82.6	2.0	1.0	97.0
independent	90	19.7	12.6	67.7	19.9	0.8	79.3
0.20	90	15.8	14.3	69.8	22.1	1.2	76.7
0.15	90	11.4	13.0	75.6	13.1	0.4	86.6
0.10	90	4.9	14.5	80.6	5.9	0.2	94.0
0.05	90	1.4	16.4	82.2	1.7	0.6	97.8
independent	200	18.5	11.6	69.9	19.8	0.5	79.7
0.20	200	18.9	13.4	67.7	20.1	1.0	78.9
0.15	200	10.7	12.8	76.5	13.3	0.8	85.9
0.10	200	4.7	14.9	80.4	6.8	0.7	92.4
0.05	200	2.3	15.4	82.3	1.7	1.1	97.2
independent	500	19.0	12.2	68.8	19.6	0.7	79.7
0.20	500	18.3	13.2	68.4	19.7	0.3	80.0
0.15	500	11.1	14.0	74.9	13.2	0.3	86.5
0.10	500	4.9	15.0	80.1	6.2	0.6	93.3
0.05	500	1.6	15.0	83.4	1.9	0.8	97.3
independent	1000	19.0	12.2	68.8	20.0	0.4	79.6
0.20	1000	17.6	13.0	69.4	20.3	0.6	79.2
0.15	1000	11.3	13.4	75.3	13.1	0.7	86.2
0.10	1000	5.2	14.2	80.6	6.2	0.6	93.2
0.05	1000	1.5	14.8	83.7	1.9	0.7	97.4
(D)							
independent	30	11.6	17.2	71.3	14.0	4.1	81.9
0.20	30	17.0	12.9	70.1	14.4	6.7	78.9
0.15	30	7.8	16.5	75.7	9.9	1.3	88.8
0.10	30	2.6	18.3	79.1	5.9	1.5	92.7
0.05	30	1.9	15.4	82.7	1.5	-1.2	99.6
independent	45	12.9	16.1	71.0	14.5	2.7	82.8
0.20	45	14.4	15.4	70.2	15.3	5.5	79.2
0.15	45	10.7	13.9	75.4	9.3	2.1	88.6
0.10	45	5.4	15.4	79.2	6.5	0.2	93.3
0.05	45	1.7	14.8	83.4	2.1	1.6	96.3
independent	90	14.4	15.1	70.5	13.8	2.9	83.3
0.20	90	13.3	15.5	71.2	17.4	3.6	79.0
0.15	90	9.4	14.5	76.2	9.4	2.5	88.1
0.10	90	2.9	16.5	80.6	4.3	1.3	94.4
0.05	90	1.3	14.6	84.1	1.3	-0.6	99.3
independent	200	13.6	15.1	71.3	14.4	3.1	82.5
0.20	200	15.4	16.6	68.0	16.4	3.6	80.0
0.15	200	8.2	15.6	76.2	10.0	2.0	88.0
0.10	200	3.9	14.7	81.4	4.8	1.6	93.6
0.05	200	1.0	15.7	83.3	1.4	0.7	97.9
independent	500	14.1	15.7	70.2	14.1	3.2	82.7
0.20	500	13.6	16.1	70.3	14.8	3.3	81.9
0.15	500	8.4	16.1	75.5	9.7	2.9	87.4
0.10	500	4.3	14.7	81.0	4.1	1.9	94.0
0.05	500	1.2	15.5	83.3	1.3	0.7	97.9
independent	1000	14.2	15.8	70.0	14.6	3.6	81.8
0.20	1000	13.6	15.4	71.0	14.4	3.7	82.0
0.15	1000	8.7	15.4	75.9	9.4	2.5	88.1
0.10	1000	3.9	15.0	81.1	4.2	1.6	94.2
0.05	1000	1.2	15.4	83.4	1.3	0.9	97.9
(E)							
independent	30	10.7	17.0	72.3	13.2	3.8	83.0
0.20	30	16.0	11.8	72.2	15.7	4.2	80.1
0.15	30	8.8	15.2	76.0	8.2	3.0	88.9
0.10	30	4.7	14.5	80.8	5.6	1.2	93.3
0.05	30	2.1	14.8	83.1	2.4	0.9	96.8
independent	45	12.1	16.3	71.5	15.4	4.6	80.0
0.20	45	14.9	16.9	68.2	14.1	5.5	80.4
0.15	45	9.8	14.0	76.2	10.3	3.2	86.5
0.10	45	3.9	16.2	80.0	4.4	0.6	94.9
0.05	45	1.3	16.4	82.2	1.5	0.9	97.7
independent	90	13.7	17.5	68.8	13.5	3.7	82.7
0.20	90	13.8	16.5	69.7	16.5	4.0	79.5
0.15	90	9.6	15.5	74.9	9.8	2.9	87.4
0.10	90	3.4	15.6	80.9	4.8	1.4	93.8
0.05	90	1.4	15.3	83.2	1.5	0.9	97.6
independent	200	13.7	15.8	70.5	14.5	3.5	82.0
0.20	200	15.0	15.7	69.3	16.5	3.8	79.7
0.15	200	8.4	15.8	75.9	10.0	2.1	87.9
0.10	200	3.9	14.9	81.2	5.1	1.4	93.6
0.05	200	1.3	14.6	84.0	1.2	1.0	97.8
independent	500	14.0	15.4	70.6	14.5	3.5	82.0
0.20	500	13.5	15.9	70.6	15.2	3.8	81.0
0.15	500	8.6	15.5	75.9	9.9	2.7	87.4
0.10	500	4.1	14.9	81.0	4.2	1.7	94.1
0.05	500	1.2	15.3	83.5	1.4	0.7	97.9
independent	1000	14.1	15.4	70.5	14.5	3.7	81.7
0.20	1000	13.3	15.8	70.8	14.4	3.8	81.8
0.15	1000	8.3	16.1	75.6	9.1	2.7	88.2
0.10	1000	3.6	15.2	81.1	4.5	1.6	93.8
0.05	1000	1.0	15.4	83.6	1.5	0.9	97.6

The percentage values for the partitioning of variance in Model A were very similar to the reference values (Table 1A) for both the patch sample sizes of five (Table 1B) and 10 (Table 1C), even when as few as 30 marker loci were used. For example, with 30 marker loci, five individuals sampled per patch, independent region-level allele frequencies, and without background migration there was 20.9% of the variance at the among-region level, 13.1% at the among-patch level, and 66.0% (Table 1B) at the within-patch level compared to 19.1%, 11.9%, and 69.0% for the corresponding reference data set (Table 1A). Sampling more individuals per patch (10 vs. five) and using higher numbers of markers brought the Model A variance partitioning percentages closer to those of the reference data sets. With 500 marker loci, 10 individuals sampled per patch, independent region-level allele frequencies, and without background migration the variance percentages differed by no more than 0.3% (Table 1C) from those of the corresponding reference data set values (Table 1A). The three trends observed in the reference data sets were also observed in the Model A data sets (Table 1B and C).

In contrast to Model A, the Model B results differed more widely from those of the reference values. The among-region variance proportions were consistently lower than the reference values, while the among-patch values were consistently higher. The within-patch variance proportions were very similar to those of the reference values. For example, with 90 marker loci and 10 individuals sampled per patch, *σ*_reg_ = 0.2 and no background migration, the among-region variance accounted for 13.8% of the total, 16.5% of the among-patch variance, and 69.7% of the within-patch variance (Table 1E) compared to the reference values of 18.2%, 12.4%, and 69.4%, respectively (Table 1A). Using more markers did not fully correct this bias. With 1000 loci, 10 individuals sampled per patch, no background migration, and *σ*_reg_ = 0.2, the among-region variance was 13.3% of the total, among-patch was 15.8%, while within patch was 70.8% (Table 1E) compared to reference values of 18.2%, 12.4%, and 69.4%, respectively (Table 1A).

The reference values for the Φ-statistics indicate that independent or distantly related regions (independent or *σ*_reg_ = 0.2) are differentiated from one another (Φ_SC_ = 0.178–0.202, Table 2A) with or without background migration. Without background migration, patches within regions are less differentiated than among regions (Φ_SC_ = 0.150–0.157), while they are not differentiated with background migration (Φ_SC_ = 0.007). Patches, disregarding regional structure, are more differentiated from each other with the presence of background migration (Φ_ST_ = 0.307–0.311) compared to patches without (Φ_ST_ = 0.205–0.208; Table 2A). Moving from independent to *σ*_reg_ = 0.05, the regional differentiation decreases from Φ_CT_ = 0.189 to Φ_CT_ = 0.015 without background migration and from Φ_CT_ = 0.202 to Φ_CT_ = 0.019 with background migration (Table 2A) while Φ_ST_ also decreases from Φ_ST_ = 0.311 to Φ_ST_ = 0.164 without background migration and from Φ_ST_ = 0.205 to Φ_ST_ = 0.025 with background migration. The Φ_SC_ remains relatively constant as *σ*_reg_ varies, but the differentiation of patches within regions is much lower for simulations with background migration (Φ_SC_ = 0.006–0.010) than simulations without (Φ_SC_ = 0.150–0.157).

**Table 2:** AMOVA Φ-statistical analyses from a simulated study of the (A) reference populations (1000 marker loci, 150 individuals sampled per patch); (B, C) for equal sampling (Model A) with five and 10 individuals sampled per patch, respectively; (D, E) for unequal sampling (Model B) with five and 10 individuals sampled per patch, respectively

*σ*_reg_	No. of markers	No background migration			Background migration		
	
		Φ_CT_	Φ_SC_	Φ_ST_	Φ_CT_	Φ_SC_	Φ_ST_
(A)							
independent	1000	0.189	0.150	0.311	0.200	0.007	0.205
0.2	1000	0.178	0.157	0.307	0.202	0.007	0.208
0.15	1000	0.110	0.150	0.250	0.140	0.010	0.130
0.1	1000	0.052	0.153	0.197	0.061	0.007	0.067
0.05	1000	0.015	0.151	0.164	0.019	0.006	0.025
(B)							
independent	30	0.209	0.165	0.340	0.176	-0.002	0.174
0.2	30	0.165	0.175	0.311	0.233	0.022	0.250
0.15	30	0.088	0.170	0.243	0.131	0.001	0.132
0.1	30	0.053	0.140	0.185	0.060	0.018	0.077
0.05	30	0.033	0.166	0.194	0.027	-0.007	0.020
independent	45	0.203	0.131	0.307	0.192	0.019	0.207
0.2	45	0.153	0.177	0.302	0.221	0.007	0.226
0.15	45	0.106	0.150	0.240	0.133	0.013	0.144
0.1	45	0.047	0.154	0.195	0.069	0.022	0.089
0.05	45	0.010	0.159	0.168	0.011	0.020	0.031
independent	90	0.204	0.162	0.333	0.193	0.009	0.201
0.2	90	0.166	0.166	0.304	0.215	-0.005	0.211
0.15	90	0.109	0.160	0.252	0.136	0.006	0.140
0.1	90	0.052	0.143	0.187	0.063	0.016	0.077
0.05	90	0.014	0.164	0.176	0.020	0.000	0.021
independent	200	0.190	0.157	0.317	0.197	0.004	0.200
0.2	200	0.191	0.159	0.320	0.208	0.010	0.216
0.15	200	0.111	0.164	0.257	0.126	0.011	0.136
0.1	200	0.048	0.147	0.188	0.070	0.010	0.079
0.05	200	0.019	0.158	0.174	0.014	0.015	0.029
independent	500	0.191	0.148	0.311	0.199	0.005	0.203
0.2	500	0.178	0.155	0.306	0.198	0.011	0.207
0.15	500	0.113	0.153	0.249	0.134	0.006	0.139
0.1	500	0.051	0.152	0.195	0.066	0.004	0.070
0.05	500	0.015	0.152	0.164	0.018	0.006	0.023
independent	1000	0.191	0.150	0.312	0.198	0.008	0.204
0.2	1000	0.179	0.160	0.310	0.203	0.009	0.211
0.15	1000	0.113	0.153	0.248	0.131	0.008	0.138
0.1	1000	0.051	0.153	0.196	0.061	0.003	0.064
0.05	1000	0.012	0.152	0.162	0.020	0.008	0.027
(C)							
independent	30	0.215	0.145	0.329	0.181	0.013	0.192
0.2	30	0.178	0.158	0.308	0.233	-0.002	0.232
0.15	30	0.102	0.151	0.237	0.128	0.008	0.135
0.1	30	0.042	0.142	0.177	0.073	0.016	0.087
0.05	30	0.010	0.157	0.166	0.015	0.009	0.024
independent	45	0.196	0.155	0.320	0.193	0.018	0.207
0.2	45	0.156	0.173	0.301	0.212	0.008	0.219
0.15	45	0.092	0.149	0.227	0.132	0.010	0.140
0.1	45	0.055	0.149	0.196	0.067	-0.002	0.065
0.05	45	0.016	0.160	0.174	0.020	0.010	0.030
independent	90	0.197	0.157	0.323	0.199	0.010	0.207
0.2	90	0.159	0.170	0.302	0.221	0.015	0.233
0.15	90	0.114	0.147	0.244	0.131	0.004	0.134
0.1	90	0.049	0.153	0.194	0.059	0.002	0.060
0.05	90	0.014	0.166	0.178	0.017	0.006	0.022
independent	200	0.185	0.143	0.301	0.198	0.006	0.203
0.2	200	0.189	0.165	0.323	0.201	0.013	0.211
0.15	200	0.108	0.143	0.235	0.133	0.009	0.141
0.1	200	0.047	0.156	0.196	0.068	0.008	0.076
0.05	200	0.024	0.157	0.177	0.018	0.011	0.028
independent	500	0.190	0.151	0.312	0.196	0.009	0.203
0.2	500	0.183	0.162	0.316	0.197	0.004	0.201
0.15	500	0.111	0.157	0.251	0.132	0.004	0.135
0.1	500	0.049	0.158	0.199	0.062	0.006	0.067
0.05	500	0.016	0.152	0.166	0.019	0.008	0.027
independent	1000	0.190	0.151	0.312	0.200	0.005	0.204
0.2	1000	0.176	0.157	0.306	0.203	0.007	0.208
0.15	1000	0.113	0.151	0.247	0.131	0.008	0.138
0.1	1000	0.052	0.150	0.194	0.062	0.006	0.068
0.05	1000	0.015	0.151	0.164	0.019	0.007	0.026
(D)							
independent	30	0.116	0.194	0.288	0.140	0.048	0.181
0.2	30	0.170	0.156	0.299	0.144	0.078	0.211
0.15	30	0.078	0.179	0.243	0.099	0.015	0.112
0.1	30	0.026	0.188	0.209	0.059	0.015	0.073
0.05	30	0.019	0.157	0.173	0.016	-0.012	0.004
independent	45	0.129	0.185	0.290	0.145	0.031	0.172
0.2	45	0.144	0.180	0.298	0.153	0.065	0.208
0.15	45	0.107	0.156	0.247	0.093	0.023	0.114
0.1	45	0.054	0.163	0.208	0.065	0.002	0.067
0.05	45	0.017	0.151	0.166	0.021	0.016	0.037
independent	90	0.144	0.177	0.295	0.138	0.034	0.167
0.2	90	0.133	0.179	0.288	0.174	0.044	0.211
0.15	90	0.094	0.160	0.239	0.094	0.028	0.119
0.1	90	0.030	0.170	0.194	0.043	0.013	0.056
0.05	90	0.013	0.148	0.159	0.013	-0.006	0.007
independent	200	0.136	0.175	0.287	0.144	0.036	0.175
0.2	200	0.154	0.196	0.320	0.164	0.044	0.200
0.15	200	0.082	0.170	0.238	0.100	0.022	0.120
0.1	200	0.039	0.153	0.186	0.048	0.016	0.064
0.05	200	0.010	0.159	0.167	0.014	0.007	0.021
independent	500	0.141	0.183	0.298	0.141	0.037	0.173
0.2	500	0.136	0.186	0.297	0.149	0.039	0.181
0.15	500	0.085	0.176	0.245	0.097	0.032	0.126
0.1	500	0.043	0.154	0.190	0.041	0.020	0.060
0.05	500	0.012	0.157	0.167	0.013	0.008	0.021
independent	1000	0.142	0.184	0.300	0.146	0.042	0.182
0.2	1000	0.136	0.178	0.290	0.144	0.043	0.180
0.15	1000	0.087	0.169	0.241	0.094	0.028	0.119
0.1	1000	0.039	0.157	0.189	0.042	0.017	0.058
0.05	1000	0.012	0.156	0.166	0.013	0.009	0.021
(E)							
independent	30	0.107	0.191	0.277	0.132	0.043	0.170
0.2	30	0.160	0.141	0.278	0.157	0.050	0.199
0.15	30	0.088	0.167	0.240	0.082	0.032	0.111
0.1	30	0.047	0.152	0.192	0.056	0.012	0.067
0.05	30	0.021	0.151	0.169	0.024	0.009	0.032
independent	45	0.122	0.186	0.285	0.154	0.055	0.201
0.2	45	0.149	0.199	0.318	0.141	0.064	0.196
0.15	45	0.098	0.155	0.238	0.103	0.035	0.135
0.1	45	0.039	0.168	0.201	0.044	0.007	0.051
0.05	45	0.013	0.167	0.178	0.015	0.009	0.024
independent	90	0.137	0.203	0.312	0.135	0.043	0.173
0.2	90	0.138	0.191	0.303	0.165	0.048	0.205
0.15	90	0.096	0.172	0.251	0.098	0.032	0.126
0.1	90	0.035	0.162	0.191	0.048	0.015	0.063
0.05	90	0.014	0.156	0.168	0.015	0.009	0.024
independent	200	0.137	0.183	0.295	0.145	0.041	0.180
0.2	200	0.150	0.185	0.308	0.165	0.046	0.203
0.15	200	0.084	0.172	0.241	0.100	0.023	0.121
0.1	200	0.039	0.155	0.188	0.051	0.014	0.064
0.05	200	0.014	0.148	0.160	0.012	0.010	0.022
independent	500	0.140	0.179	0.294	0.145	0.041	0.180
0.2	500	0.135	0.184	0.294	0.152	0.045	0.190
0.15	500	0.086	0.170	0.241	0.099	0.030	0.126
0.1	500	0.041	0.156	0.190	0.042	0.017	0.059
0.05	500	0.012	0.155	0.165	0.014	0.007	0.021
independent	1000	0.141	0.179	0.295	0.145	0.044	0.183
0.2	1000	0.133	0.183	0.292	0.144	0.044	0.182
0.15	1000	0.083	0.175	0.244	0.091	0.030	0.118
0.1	1000	0.036	0.158	0.189	0.045	0.017	0.062
0.05	1000	0.010	0.155	0.164	0.015	0.009	0.024

In data sets for Model A, Φ statistics were very close to the reference values with as few as 30 markers for patches sampled with five or 10 individuals (Table 2B and C). For example, with 30 markers and 10 samples per patch the Φ_CT_ values were 0.21, 0.18, and 0.04 for independent region-level allele frequencies, *σ*_reg_ = 0.2, or *σ*_reg_ = 0.1 (Table 2C) compared with reference values of 0.19, 0.18, and 0.05, respectively (Table 2A). With 200 marker loci, Φ statistics differed by not more than 0.01 from the reference values both with and without background migration with 10 individuals sampled per patch (Table 2B and C). In Model B, the among-region genetic differentiation, Φ_CT_, was consistently underestimated while the among-patch within-region patch differentiation, Φ_SC_ was consistently overestimated (Table 2D and E). For example, with 45 markers, no background migration, five individuals sampled per population, and independent region-level allele frequencies, Φ_CT_ = 0.13, Φ_SC_ = 0.19, and Φ_ST_ = 0.29 (Table 2D) compared to Φ_CT_ = 0.19, Φ_SC_ = 0.15, and Φ_ST_ = 0.31 for the reference values (Table 2A). In Model B, the Φ_ST_ values closely matched those of the reference data sets.

### Structure

**Figure 5:**
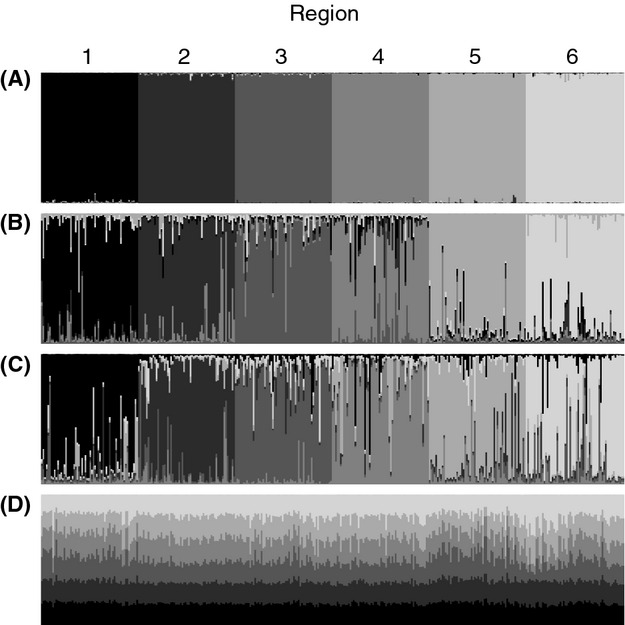
Performance of STRUCTURE's grouping algorithm with equal sampling (Model A), with vertical lines (individual genotypes) and different colors (proportional regional membership) being are assigned by STRUCTURE: (A) excellent resolution of regions, utilizing 1000 marker loci with ten individuals sampled per patch and allele frequencies independent among regions without background migration; (B) poorer resolution when using 45 markers (all other settings identical to [A]); (C) poorer resolution using 30 marker loci (all other settings identical to [A]); (D) a failure to resolve different regions when using only 30 marker loci with more closely related regions (*σ*_reg_ = 0.1) (same no. of individuals sampled/patch and no background migration). The parameter *σ*_reg_ is a measure of how closely related the allele frequencies are among regions. Low values indicate greater similarity.

Two trends were apparent in the STRUCTURE plots for Model A. First, using a large number of marker loci provided the best resolution of regions. For example, with 1000 markers, no background migration and independent region-level allele frequencies, individuals (vertical lines) were correctly assigned to their respective regions, as shown by the crisp separation of shades (Fig. 5A). As the number of markers decreased, individuals were not as clearly assigned to the correct group (shown by having multiple shades within a vertical line) as with 45 markers, independent region-level allele frequencies, and without background migration (Fig. 5B). The second trend was that individuals were most clearly resolved into the correct regions when regions were independent or distantly related (region-level allele frequencies independent or *σ*_reg_ = 0.2). Using 30 markers, independent region-level allele frequencies, and without background migration the regions were still somewhat resolved (Fig. 5C), but regions were not resolved at all with 30 markers, *σ*_reg_ = 0.1, and no background migration (Fig. 5D). The presence of background migration had little effect on the grouping except for the closely related regions where the presence of background migration appeared to make resolution more difficult (Figs. S1A–F, S2A–F). Having a larger number of individuals sampled per patch (10 as opposed to five), increased the resolution of regions in STRUCTURE (compare Figs. S1A–F, S2A–F).

**Figure 6:**
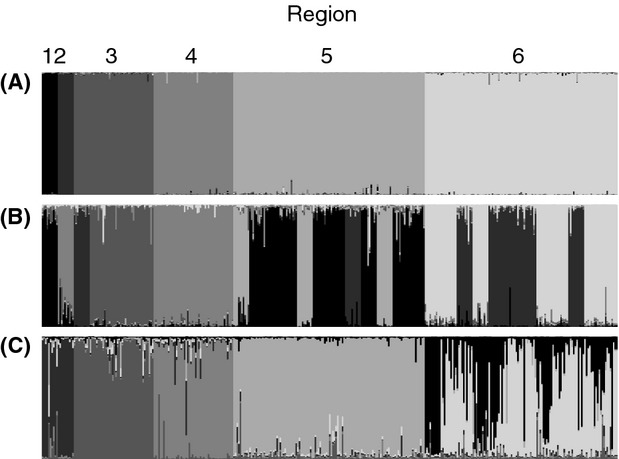
Performance of STRUCTURE's grouping algorithm with unequal sampling (Model B), with vertical lines (individual genotypes) and different colors (proportional regional membership) being assigned by 598 STRUCTURE: (A) the inability to resolve all regions with ten individuals sampled per patch, no background migration, allele frequencies independent among regions, and 1000 loci; (B) closely related regions (*σ*_reg_ = 0.1) prevented correct grouping of all genotypes; (C) under-sampled regions (regions 1 and 2) grouped together. The parameter *σ*_reg_ is a measure of how closely related the allele frequencies are among regions. Low values indicate greater similarity.

As in Model A, in Model B increasing the number of marker loci and having more distantly related regions (higher values of *σ*_reg_) increased the resolution (Figs. S3A–F, S4A–F). The best resolution was achieved with 1000 marker loci, 10 individuals sampled per patch, and independent regions (Fig. 6A). Additionally, regions in simulations with more closely related regions were difficult to resolve as illustrated by the simulation with 1000 marker loci, 10 individuals sampled per patch, no background migration and closely related regions (*σ*_reg_ = 0.05; Fig. 6B). A common pattern with unequal sampling (Model B) was to have the two least sampled regions (regions 1 and 2) incorrectly grouped together as shown in the simulation with 90 marker loci, no background migration, and *σ*_reg_ = 0.2 (Fig. 6C).

The effect of background migration was complex. In some cases regions were better resolved without background migration. For example in Model B with 90 markers, 10 individuals per patch, and *σ*_reg_ = 0.1 (Fig. S4C). In several cases the regions were more correctly resolved with background migration (Fig. S4E, 500 markers, *σ*_reg_ = 0.1, 0.15), but for most combinations of markers and *σ*_reg_ the results were similar with and without background migration.

**Figure 7:**
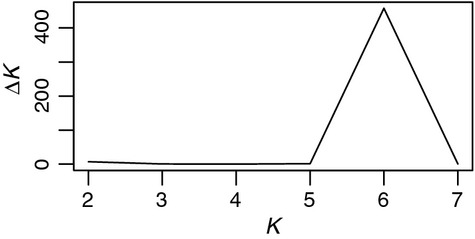
Linear plot of ΔK, producing a large peak at the correct value of K (K = 6), the most likely number of genetically distinct clusters (with 200 marker loci, ten individuals sampled per patch, independent regions, without background migration).

For Model A, using the ΔK method to determine the correct number of regions was most successful when regions were independent and a large number of markers were used. For example, with 10 individuals sampled per patch, independent region-level allele frequencies, and 200 loci, there is a large peak at K = 6 (Fig. 7). The ΔΚ method failed to detect the correct number of regions, indicated by the primary peak on the plot not falling on the point K = 6, for highly related data sets (*σ*_reg_ ≤ 0.1) with no background migration when fewer than 90 markers were used with 10 samples per patch, and fewer than 500 markers when only five individuals were sampled per patch (Figs. S5A–F, S6A–F). The independent and distantly related data sets without background migration had peaks at K = 6 for all numbers of markers when 10 individuals were sampled per patch, however many plots had secondary peaks at smaller K values (Figs. S5A–F, S6A–F). Unlike in Model A, with Model B using the ΔK method, we were unable to determine the correct number of regions in all but a few of the data sets (Figs. S7A–F, S8A–F). The ΔK method underestimated the true value of K in most analyses for Model B (Figs. S7A–F, S8A–F).

### Among-region migration

**Figure 8:**
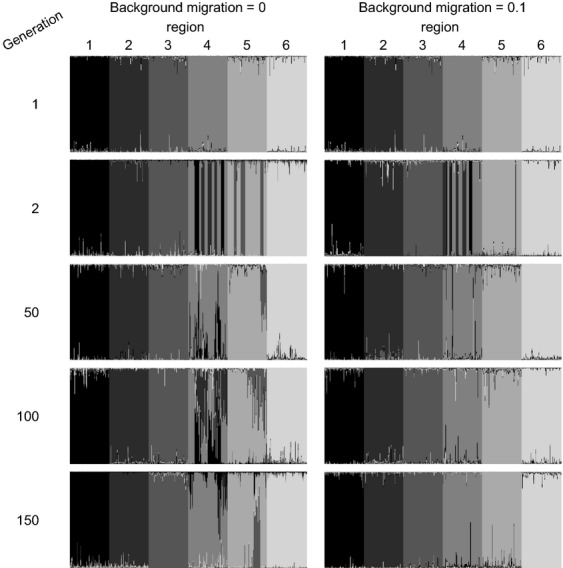
The effect of migration on the STRUCTURE analyses with an among-region migration event occurring between generations one and two. Migrants are clearly visible in regions four and five as shown by some vertical lines in the immigrant regions (regions four and five) in the generation two plots, having the same shade as the emigrant regions (regions one, two, and three). The immigrant signature is diminished by generation 50 with background migration (second column) while immigrant or hybrid genotypes are visible through generation 150 (without background migration, first column).

When among-region migration occurred (Model C), the presence of background migration had a large effect on how long admixed individuals were detected using STRUCTURE. All six regions were clearly resolved in STRUCTURE prior to among-region migration in generation one both with and without background migration (Fig. 8). In generation two, just after the among-region migrations, individuals from regions one and two were clearly discerned in region four and genotypes from region three were visible in region five both with and without background migration. Admixed individuals were resolved through generation 150 without background migration (Fig. 8). When background migration was present, only a few admixed individuals were resolved after 50 generations with among-region migration in regions four and five.

### Small sample sizes

Sampling only five individuals (as opposed to 10) from each patch produced only slightly different results for both the Φ-statistics and variance partitioning within the AMOVAs. For example, in Model A using 30 loci, without background migration and independent regions, the small sample data sets had 20.9% variance among regions, 13.1% among patches, and 66.0% within patches (Table 1B) versus 21.5%, 11.4%, and 67.1% for the data sets with 10 individuals sampled per patch (Table 1C). These proportions differed only slightly from those of the corresponding reference data set (Table 1A). Increasing the number of loci decreased the differences so that the Model A data set with five individuals sampled per patch, independent regions, no background migration, and 1000 loci differed by not more than 0.1% in any of the variance components from the corresponding data set with 10 individuals sampled per patch. The STRUCTURE results were generally similar with five and 10 individuals sampled per patch, however sampling 10 individuals per patch produced slightly better resolution of distinct regions when the number of loci was small (compare Figs. S1A and S2A), or the regions were closely related (compare Figs. S1E and S2E, *σ*_reg_ = 0.05).

## Discussion

When planning a study using dominant markers, the minimum number of markers required depends, among other factors, on the analyses being performed. Based on the AMOVAs, as few as 30 markers will yield acceptable results (Table 1B–E), but STRUCTURE will require greater numbers of markers (generally 90 or more; Figs. S1–S4). In addition to greater numbers of markers, a higher degree of differentiation among regions improved the resolution using STRUCTURE. Due to the poor performance of the ΔK method with unequal sampling, the ideal sampling scheme would sample equally from genetically distinct groups. When using STRUCTURE to infer admixture, knowledge of the amount of inter-patch migration is needed.

Equal sampling may be difficult to achieve in a study of real organisms, especially when the goal is to detect cryptic population structure, and knowledge of the level of interpatch migration may be scarce. STRUCTURE, and especially the ΔK method however, perform optimally when genetically distinct groups have been equally sampled (compare Figs. S5 and S6 to Figs. S7 and S8) and background migration hampers the ability to detect admixed individuals. It is recommended that researchers use all available demographic information when devising a sampling scheme to try to achieve equal sampling of genetically distinct groups. In *P. arundinacea*, for example, it is known that the species is native to N. America and Europe (Merigliano and Lesica 1998) with multiple introduction events from Europe to N. America (Galatowitsch et al. 1999) and that forage cultivars contain both N. American and European germplasm. In this example, a balanced strategy would be to sample equally from European and N. American wild populations and forage cultivars. As a wind-pollinated species, *P. arundinacea* may have significant gene flow mediated among patches via wind-transported pollen. This could have the same effect as background migration and may hamper the ability to detect admixed individuals.

Although this study used *P. arundinacea* as a model, future researchers desiring to implement these analysis strategies with other plant species can use our R scripts (see Data S1). This will allow for researchers to adjust many of the parameters of the models including patch size, number of individuals sampled, chromosome number/size, *σ*_reg_, migration rates, and others to match more closely their study organism.

The most critical factor in determining the number of required markers is the level of genetic differentiation among populations or regions. Because the real amount of genetic differentiation among regions and among patches within regions is initially not known, a determination of the number of markers needed should be included as part of the experimental design. The use of at least 200 markers has previously been recommended (Singh et al. 2006; Bonin et al. 2007), but the true minimum needed depends on an analysis of the genetic differentiation.

Every organism and research question may require a different number of markers to resolve the true clusters in STRUCTURE. Utilizing the fact that the AMOVA is accurate with a small number of markers, the following protocol is recommended to determine the needed quantity of markers for STRUCTURE. First, generate a modest number of markers, in the range 30–50 and perform an AMOVA. Although Φ_ST_ is not a true estimator of population differentiation (Jost 2008), it is readily calculated via AMOVA and may serve as an initial guideline. Second, to determine the number of markers needed, use the calculated value of Φ_ST_ to determine the number of markers needed for STRUCTURE. If Φ_ST_ is 0.3 or greater, adequate results can be achieved with only 45–90 loci. If Φ_ST_ is between 0.2 and 0.3, a minimum of 90 loci is needed. If Φ_ST_ is between 0.1 and 0.2, a minimum of 200 loci is recommended. Finally, if Φ_ST_ is less than 0.1, 500 or more marker loci may be required to achieve clear resolution of genetically distinct groups in STRUCTURE. If the ΔK method were used to determine the number of genetically distinct clusters, great care must be taken to sample equally from putatively distinct populations and/or regions.

## Supporting Information

**Data S1.** R Script.

**Figure S1.** The performance of STRUCTURE with five individuals sampled per patch with equal sampling among regions (model A) for all levels of *σ*_reg_, all numbers of loci, and with and without background migration is illustrated in the plots. Each vertical line represents one genotype, while the colors represent the proportional group membership coefficients assigned to each genotype by STRUCTURE. Figures S1A–F represent simulations with 30, 45, 90, 200, 500, and 1000 marker loci respectively.

**Figure S2.** The performance of STRUCTURE with ten individuals sampled per patch with equal sampling among regions (model A) for all levels of *σ*_reg_, all numbers of loci, and with and without background migration is illustrated in the plots. Each vertical line represents one genotype, while the colors represent the proportional group membership coefficients assigned to each genotype by STRUCTURE. Figures S2A–F represent simulations with 30, 45, 90, 200, 500, and 1000 marker loci respectively.

**Figure S3.** The performance of STRUCTURE with five individuals sampled per patch with unequal sampling among regions (model B) for all levels of *σ*_reg_, all numbers of loci, and with and without background migration is illustrated in the plots. Each vertical line represents one genotype, while the colors represent the proportional group membership coefficients assigned to each genotype by STRUCTURE. Figures S3A–F represent simulations with 30, 45, 90, 200, 500, and 1000 marker loci respectively.

**Figure S4.** The performance of STRUCTURE with ten individuals sampled per patch with unequal sampling among regions (model B) for all levels of *σ*_reg_, all numbers of loci, and with and without background migration is illustrated in the plots. Each vertical line represents one genotype, while the colors represent the proportional group membership coefficients assigned to each genotype by STRUCTURE. Figures S4A–F represent simulations with 30, 45, 90, 200, 500, and 1000 marker loci respectively.

**Figure S5.** The ΔK method was evaluated using all simulated data sets. The ΔK method produces a peak at the most likely number of groups (K) based on the output of the STRUCTURE simulations. A distinct peak indicates the estimated “true” K. The height of the peak can be interpreted as the degree of confidence in the estimate. For all simulations, the true value of K is 6. Figures S5A–E show the results for equal sampling (model A) with five individuals sampled per patch using 30, 45, 90, 200, 500, and 1000 marker loci.

**Figure S6.** The ΔK method was evaluated using all simulated data sets. The ΔK method produces a peak at the most likely number of groups (K) based on the output of the STRUCTURE simulations. A distinct peak indicates the estimated “true” K. The height of the peak can be interpreted as the degree of confidence in the estimate. For all simulations, the true value of K is 6. Figures S6A–E show the results for equal sampling (model A) with ten individuals sampled per patch using 30, 45, 90, 200, 500, and 1000 marker loci.

**Figure S7.** The ΔK method was evaluated using all simulated data sets. The ΔK method produces a peak at the most likely number of groups (K) based on the output of the STRUCTURE simulations. A distinct peak indicates the estimated “true” K. The height of the peak can be interpreted as the degree of confidence in the estimate. For all simulations, the true value of K is 6. Figures S7A–E show the results for unequal sampling (model B) with five individuals sampled per patch using 30, 45, 90, 200, 500, and 1000 marker loci.

**Figure S8.** The ΔK method was evaluated using all simulated data sets. The ΔK method produces a peak at the most likely number of groups (K) based on the output of the STRUCTURE simulations. A distinct peak indicates the estimated “true” K. The height of the peak can be interpreted as the degree of confidence in the estimate. For all simulations, the true value of K is 6. Figures S8A–E show the results for unequal sampling (model B) with ten individuals sampled per patch using 30, 45, 90, 200, 500, and 1000 marker loci.

## References

[b1] Baird NA, Etter PD, Atwood TS, Currey MC, Shiver AL, Lewis ZA (2008). Rapid SNP discovery and genetic mapping using sequenced RAD markers. PLoS ONE.

[b2] Balloux F (2001). EASYPOP (Version 1.7): a computer program for population genetics simulations. J. Hered.

[b3] Bezault E, Mwaiko S, Seehausen O (2011). Population genomic tests of models of adaptive radiation in Lake Victoria region cichlid fish. Evolution.

[b4] Bonin A, Ehrich D, Manel S (2007). Statistical analysis of amplified fragment length polymorphism data: a toolbox for molecular ecologists and evolutionists. Mol. Ecol.

[b5] Cavers S, Degen B, Caron H, Lemes MR, Margis R, Salgueiro F (2005). Optimal sampling strategy for estimation of spatial genetic structure in tree populations. Heredity.

[b6] Culley TM, Wolfe AD (2001). Population genetic structure of the cleistogamous plant species *Viola pubescens* Aiton (Violaceae), as indicated by allozyme and ISSR molecular markers. Heredity.

[b7] Dray S, Dufour AB (2007). The ade4 package: implementing the duality diagram for ecologists. J. Stat. Softw.

[b8] Evanno G, Regnaut S, Goudet J (2005). Detecting the number of clusters of individuals using the software structure: a simulation study. Mol. Ecol.

[b9] Excoffier L, Smouse PE, Quattro JM (1992). Analysis of molecular variance inferred from metric distances among DNA haplotypes: application to human mitochondrial DNA restriction data. Genetics.

[b10] Falush D, Stephens M, Pritchard JK (2007). Inference of population structure using multilocus genotype data: dominant markers and null alleles. Mol. Ecol. Notes.

[b11] Galatowitsch SM, Anderson NO, Ascher P (1999). Invasiveness in wetland plants in temperate North America. Wetlands.

[b12] Hollingsworth PM, Ennos RA (2004). Neighbour joining trees, dominant markers and population genetic structure. Heredity.

[b13] Jakubowski AR, Casler MD, Jackson RD (2013). Genetic evidence suggests a widespread distribution of native North American populations of reed canarygrass. Biol. Invasions.

[b14] Jost L (2008). G_ST_ and its relatives do not measure differentiation. Mol. Ecol.

[b15] Kimura M, Weiss GH (1964). The stepping stone model of population structure and the decrease of genetic correlation with distance. Genetics.

[b16] McWilliam JR, Neal-Smith CA (1962). Tetraploid and hexaploid chromosome races of *Phalaris arundinacea* L. Crop Pasture Sci.

[b17] Meekins JF, Ballard HE, McCarthy BC (2001). Genetic variation and molecular biogeography of a North American invasive plant species (*Alliaria petiolata*, Brassicaceae). Int. J. Plant Sci.

[b18] Merigliano MF, Lesica P (1998). The native status of reed canarygrass (*Phalaris arundinacea* L.) in the Inland Northwest, USA. Nat. Area. J.

[b19] Nelson MF, Anderson NO, Casler MD, Jakubowski AR (2013). Population genetic structure of N. American and European *Phalaris arundinacea* L. as inferred from inter-simple sequence repeat markers. Biol. Invasions.

[b20] Neuenschwander S, Hospital F, Guillaume F, Goudet J (2008). quantiNemo: an individual-based program to simulate quantitative traits with explicit genetic architecture in a dynamic metapopulation. Bioinformatics.

[b21] Nybom H (2004). Comparison of different nuclear DNA markers for estimating intraspecific genetic diversity in plants. Mol. Ecol.

[b22] Pritchard JK, Stephens M, Donnelly P (2000). Inference of population structure using multilocus genotype data. Genetics.

[b23] R Development Core Team (2011). R: a language and environment for statistical computing.

[b24] Schmidt K, Jensen K (2000). Genetic structure and AFLP variation of remnant populations in the rare plant *Pedicularis palustris* (Scrophulariaceae) and its relation to population size and reproductive components. Am. J. Bot.

[b25] Singh M, Chabane K, Valkoun J, Blake T (2006). Optimum sample size for estimating gene diversity in wild wheat using AFLP markers. Genet. Resour. Crop Evol.

[b26] Vos P, Hogers R, Bleeker M, Reijans M, Lee TVD, Hornes M (1995). AFLP: a new technique for DNA fingerprinting. Nucleic Acids Res.

[b27] Weimarck A (1968). Self-incompatibility in the Gramineae. Hereditas.

[b28] Wolfe AD, Xiang Q, Kephart SR (1998). Assessing hybridization in natural populations of *Penstemon* (Scrophulariaceae) using hypervariable intersimple sequence repeat (ISSR) bands. Mol. Ecol.

[b29] Zietkiewicz E, Rafalski A, Labuda D (1994). Genome fingerprinting by simple sequence repeat (SSR)-anchored polymerase chain reaction amplification. Genomics.

