# Supplementary material for: How many marker loci are necessary? Analysis of dominant marker data sets using two popular population genetic algorithms
Source: Ecol Evol. 2013 Aug 28;3(10):3455–70. doi: 10.1002/ece3.725 (PMC3797491; doi:10.1002/ece3.725)
Supplement: Supplementary file 2 [file ece30003-3455-SD2.docx]

Supplemental Figure 1. The performance of STRUCTURE with five individuals sampled per patch with equal sampling among regions (model A) for all levels of $\sigma_{reg}$, all numbers of loci, and with and without background migration is illustrated in the plots. Each vertical line represents one genotype, while the colors represent the proportional group membership coefficients assigned to each genotype by STRUCTURE. Supplemental Figures 1A – 1F represent simulations with 30, 45, 90, 200, 500, and 1000 marker loci respectively.

Supplemental Figure 2. The performance of STRUCTURE with ten individuals sampled per patch with equal sampling among regions (model A) for all levels of $\sigma_{reg}$, all numbers of loci, and with and without background migration is illustrated in the plots. Each vertical line represents one genotype, while the colors represent the proportional group membership coefficients assigned to each genotype by STRUCTURE. Supplemental Figures 2A – 2F represent simulations with 30, 45, 90, 200, 500, and 1000 marker loci respectively.

Supplemental Figure 3. The performance of STRUCTURE with five individuals sampled per patch with unequal sampling among regions (model B) for all levels of $\sigma_{reg}$, all numbers of loci, and with and without background migration is illustrated in the plots. Each vertical line represents one genotype, while the colors represent the proportional group membership coefficients assigned to each genotype by STRUCTURE. Supplemental Figures 3A – 3F represent simulations with 30, 45, 90, 200, 500, and 1000 marker loci respectively.

Supplemental Figure 4. The performance of STRUCTURE with ten individuals sampled per patch with unequal sampling among regions (model B) for all levels of $\sigma_{reg}$, all numbers of loci, and with and without background migration is illustrated in the plots. Each vertical line represents one genotype, while the colors represent the proportional group membership coefficients assigned to each genotype by STRUCTURE. Supplemental Figures 4A – 4F represent simulations with 30, 45, 90, 200, 500, and 1000 marker loci respectively.

Supplemental Figure 5. The ΔK method was evaluated using all simulated data sets. The ΔK method produces a peak at the most likely number of groups (K) based on the output of the STRUCTURE simulations. A distinct peak indicates the estimated ‘true’ K. The height of the peak can be interpreted as the degree of confidence in the estimate. For all simulations, the true value of K is 6. Supplemental figures 5A – 5E show the results for equal sampling (model A) with five individuals sampled per patch using 30, 45, 90, 200, 500, and 1000 marker loci.

Supplemental Figure 6. The ΔK method was evaluated using all simulated data sets. The ΔK method produces a peak at the most likely number of groups (K) based on the output of the STRUCTURE simulations. A distinct peak indicates the estimated ‘true’ K. The height of the peak can be interpreted as the degree of confidence in the estimate. For all simulations, the true value of K is 6. Supplemental figures 6A – 6E show the results for equal sampling (model A) with ten individuals sampled per patch using 30, 45, 90, 200, 500, and 1000 marker loci.

Supplemental Figure 7. The ΔK method was evaluated using all simulated data sets. The ΔK method produces a peak at the most likely number of groups (K) based on the output of the STRUCTURE simulations. A distinct peak indicates the estimated ‘true’ K. The height of the peak can be interpreted as the degree of confidence in the estimate. For all simulations, the true value of K is 6. Supplemental figures 7A – 7E show the results for unequal sampling (model B) with five individuals sampled per patch using 30, 45, 90, 200, 500, and 1000 marker loci.

Supplemental Figure 8 The ΔK method was evaluated using all simulated data sets. The ΔK method produces a peak at the most likely number of groups (K) based on the output of the STRUCTURE simulations. A distinct peak indicates the estimated ‘true’ K. The height of the peak can be interpreted as the degree of confidence in the estimate. For all simulations, the true value of K is 6. Supplemental figures 8A – 8E show the results for unequal sampling (model B) with ten individuals sampled per patch using 30, 45, 90, 200, 500, and 1000 marker loci.

Figure S1a

Figure S1b

Figure S1c

Figure S1d

Figure S1e

Figure S1f

Figure S2a

Figure S2b

Figure S2c

Figure S2d

Figure S2e

Figure S2f

Figure S3a

Figure S3b

Figure S3c

Figure S3d

Figure S3e

Figure S3f

Figure S4a

Figure S4b

Figure S4c

Figure S4d

Figure S4e

Figure S4f

Figure S5a

Figure S5b

Figure S5c

(Model A – 90 loci – 5 samples/population)

Figure S5d

(Model A – 200 loci – 5 samples/population)

Figure S5e

(Model A – 500 loci – 5 samples/population)

Figure S5f

(Model A – 1000 loci – 5 samples/population)

Figure S6a

Figure S6b

Figure S6c

Figure S6d

Figure S6e

Figure S6f

Figure S7a

Figure S7b

Figure S7c

Figure S7d

Figure S7e

Figure S7f

Figure S8a

Figure S8b

Figure S8c

Figure S8d

Figure S8e

Figure S8f
